# Molecular Docking and Molecular Dynamics Simulation of Fisetin, Galangin, Hesperetin, Hesperidin, Myricetin, and Naringenin against Polymerase of Dengue Virus

**DOI:** 10.1155/2022/7254990

**Published:** 2022-03-20

**Authors:** Jaka Fajar Fatriansyah, Raihan Kenji Rizqillah, Muhammad Yusup Yandi

**Affiliations:** Department of Metallurgical and Materials Engineering, Faculty of Engineering, University of Indonesia, Depok 16424, Jawa Barat, Indonesia

## Abstract

Dengue fever is a disease spread by the DENV virus through mosquitoes. This disease is dangerous because there is no specific drug, vaccine, or antiviral against the DENV virus, insisting on drug discovery for dengue fever. RNA-dependent RNA polymerase (RdRp) enzyme in DENV can be a drug target because it has an important role in the virus replication process. In this research, in silico simulations were carried out on bioflavonoid compounds, namely, Fisetin, Galangin, Hesperetin, Hesperidin, Myricetin, and Naringenin with Quercetin as control ligand. QSAR analysis showed that all ligand has the probability to be antiviral and RNA synthesis inhibitor. Docking scores showed that Myricetin, Hesperidin, and Fisetin show strong performance while Hesperidin, Hesperetin, and Naringenin showed strong performance in MM/GBSA. Only Hesperidin showed strong performance in both scorings. Further investigation by ADMET analysis was done to investigate toxicology and pharmacological properties. Our molecular dynamics study through RMSD showed that even though Quercetin does not give good scoring values in both docking score and MM/GBSA, it has robust stable interaction to RdRp. The strong performance of Hesperidin was also validated by protein-ligand contact fraction in 5 ns. Overall, we observed that Hesperidin shows good potential as a DENV-3-RdRp inhibitor in par with Quercetin, although further in vitro study should be conducted.

## 1. Introduction

Dengue fever (DF) is a disease caused by Dengue Virus (DENV), a virus that can be injected into humans through the stings of *Aedes aegypti* and *Aedes albopictus* mosquitoes. DENV is classified in the Flaviviridae family and Flavivirus genus alongside other pathogenic viruses [1]. There are four serotypes of DENV, and all of them can cause DF [2]. This disease can be lethal to the untreated patient. Currently, there is no specific antiviral to treat dengue. The patients are usually treated symptomatically and supportively with fever medicine such as paracetamol and bed rest [3]. Four genetically related DENV serotypes: DENV-1, DENV-2, DENV-3, and DENV-4 can cause DF [4]. In Indonesia, DENV-3 has been the dominant case since the great outbreak of 1988, although in the 2000s, it has been replaced with DENV-a and DENV-2 [5]. In 2019, the number of DF cases caused by DENV-3 had soared, reaching the highest number of cases in 10 years in North Sulawesi Province, Indonesia [6]. Nevertheless, DENV-3 is one of the major threats to Indonesian public health.

Plant-derived compounds such as flavonoids have attracted attention for drug and antiviral discovery due to their natural origin, low side effects, and abundance. Flavonoids are a category of low molecular polyphenolic compounds produced exclusively in plants [7]. Flavonoids have various biological and pharmacological activities, i.e., antiallergic, anti-inflammatory, antioxidant, antimicrobial (antibacterial, antifungal, and antiviral), anticancer, and antidiarrheal activity [8–11]. Several bioflavonoid compounds, such as Quercetin, Hesperetin, naringin, and daidzein, are experimentally reported to have antiviral activity against several viruses [12]. Molecular docking showed that 33 flavonoids could be used as potent dengue polymerase inhibitors [13].

The in silico study was considered as a preliminary study to determine the interaction mechanism between DENV RdRp and its inhibitor. Rdrp was chosen as a target because it has an important role in the virus replication process [14]. In this study, we conduct in silico methods, molecular docking, and molecular dynamics to assess some flavonoid ligands (Fisetin, Galangin, Hesperetin, Hesperidin, Myricetin, and Naringenin) docking score and their molecular dynamics behaviour as DENV-3 RdRp inhibitor, with Quercetin as control ligand. Molecular docking is used to analyze the model and affinity of the interaction of enzymes/proteins with ligands/inhibitors, while molecular dynamics is used to observe the stability of the bonds in a dynamics picture [15–17], in contrast with docking, which is a static picture. The molecular dynamics method consists of the numerical, step-by-step solution of the Newtonian classical equations of motion [15].

In this article, we examine the dynamics of interaction between inhibitors and the DENV-3 RdRp enzyme. Until now, there were no complete studies in molecular dynamics of Fisetin, Galangin, Hesperetin, Hesperidin, Myricetin, and Naringenin against the target receptor of DENV RdRp, though the study in molecular docking is abundant. Using the molecular dynamics approach, we expect to see a complete picture of ligand-target interaction, which may give insight into the new drug/treatment discovery for DF disease.

## 2. Computational Methods

### 2.1. Ligand and Protein Preparation

**Figure 1:**
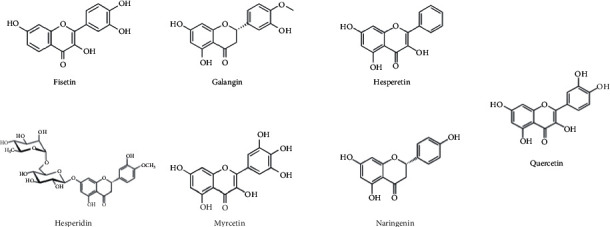
Chemical structure of Fisetin, Galangin, Hesperetin, Hesperidin, Myricetin, Naringenin, and Quercetin.

Maestro Schrödinger was used to conduct molecular docking and molecular dynamics simulations. The crystal structure of DENV-3 Rdrp was retrieved from the RCSB protein data bank (PDB ID : 3VWS), where its structure was determined by X-ray diffraction with a resolution of 2.1 Å. The structure of Fisetin, Galangin, Hesperetin, Hesperidin, Myricetin, and Naringenin ligands was collected from the PubChem database, as shown in Figure 1.

Rdrp as target protein/receptor was prepared using Protein Preparation Wizard, which consists of the following adopted steps from Anusuya and Gromiha [18]: (1) assignment of the bond orders; (2) addition of hydrogen atoms, missing atoms, and missing loops; (3) removal of water molecules that were not involved in interaction; (4) refining the structure using prime; (5) generating suitable ionization and tautomeric states of the hetero groups; (6) optimization of hydrogen bonds to avoid steric clashes; (7) refining the structure with restrained minimization to an RMSD of 0.3 Å using the imperf module and OPLS-2005 force field.

All ligands were prepared using LigPrep v3.1 by desalting and adding hydrogen atoms. Epik v2.9 was used to prepare all the possible ionization and tautomeric states at a pH range of 7 ± 2. Energy minimization was conducted to obtain the lowest energy conformation using the OPLS-2005 force field. After the preparation of both target protein and ligands, molecular docking was executed.

### 2.2. QSAR Analysis of Ligand Compound

Quantitative structure-activity relationship (QSAR) analysis was performed to investigate the feasibility of the ligand compounds' chemical biological activity [19]. The analysis was carried out by utilizing Prediction of Activity Spectra for Substances (PASS), web-based software, which provides predictions of a variety of biological activities based on the organic structure of the compound. PASS makes predictions of biological activities and pharmacological effects based on the chemical structure of a compound. The prediction result is in the form of probability of being active (Pa) and probability of being inactive (Pi) with values ranging from 0.0 to 1.0 [20].

### 2.3. Binding Site of Target Protein

Protein-ligand binding site is a region of the protein that binds to a ligand, usually located in a pocket-like region. This protein-ligand binding site is called an active site where the surface of this site performs protein function. The binding phenomenon of a ligand to a protein-ligand binding site usually triggers a change in the protein conformation and results in altered cellular function. Hence, binding site on a protein is a critical part of signal transduction pathways [21].

**Figure 2:**
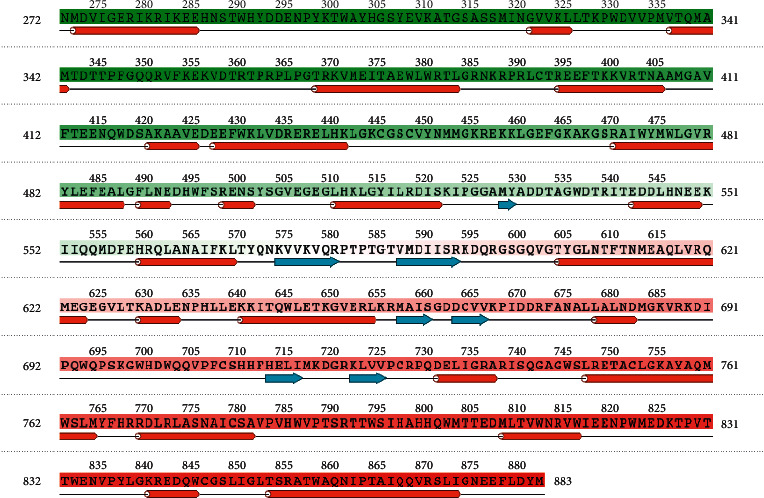
Protein sequence of Dengue Virus Serotype 3 Rdrp.

In this study, the binding site of target protein 3VWS was determined in two ways: utilizing sitemap and collecting binding site from RSCB Protein Data Bank (Table 1). Binding site data are in the form of an array of residue numbers (author's number), and the corresponding amino acid is shown in Figure 2. The binding site will be used to examine if an interaction occurs at a substantial region of the protein.

### 2.4. Molecular Docking

The grid for molecular docking was set on the centre of inhibitor NITD-107, the native ligand of 3VWS, in the active site of the protein. Van der Waals radius scaling factor was set to 1, and the partial charge cut-off was set to 0.25. All ligands were docked to target protein RdRp with the Extra Precision (XP) algorithm to avoid false positives [20].

After completing molecular docking, further calculation of molecular mechanics with generalized Born and surface area solvation (MM/GBSA) was conducted to obtain the binding free energy (Δ*G* bind) between ligands and target protein. The binding energy can be calculated as follows:

(1) $$\begin{array}{c} \Delta G_{\text{bind}}=G_{\text{complex}}-\left(G_{\text{protein}}+G_{\text{ligand}}\right), \end{array}$$

where *G*_complex_, *G*_protein_, and *G*_ligand_ are the free energies of complex, protein, and ligand, respectively.

### 2.5. ADMET Prediction

Absorption, distribution, metabolism, excretion, and toxicity (ADMET) prediction of all ligand structures were analyzed using Toxtree [22] and SWISSADME [21] server to examine the toxicology and pharmacological properties of those compounds. The observed properties are carcinogenicity, AMES Test, Lipinski's rule of five for druglikeness, mutagenicity, and corrosive properties.

### 2.6. Molecular Dynamics Simulation

The output of docked complex from XP Glide docking was used for molecular dynamics simulation. Desmond/Maestro Schrödinger's system builder was used to make a complex protein-ligand system in a water solvent system. The system shape is an orthorhombic box with a size of 10 Å, and the volume was minimized. The Simple Point Charge (SPC) water model was used to model water solvent. The docked complex was neutralized by 9 Cl ions, and 0.15 M concentration of Na+ and Cl ions was added to the system.

The entire system was relaxed at the initial simulation. Once the system was ready, it was simulated in an isothermal–isobaric (NPT) ensemble of 310 k and 1.01325 bar pressure. The molecular dynamics simulation was set to 5 ns with a timestep of 4.8 ps, generating 520 frames.

## 3. Results and Discussion

### 3.1. QSAR Analysis Result

Table 2 shows the summarized results of PASS prediction of biological activity spectra. The selected biological activities are general antiviral activity, viral entry inhibitor, and RNA synthesis inhibitor. The latter activity was specifically observed because the mechanism of the ligand prevents the virus replication process by inhibiting RdRp protein. The probability of biological activity of a ligand increases with higher values of Pa and lower values of Pi, i.e., Pa > Pi [23]. It can be seen in Table 2 that, in general, all ligands are predicted to possess antiviral activity and RNA synthesis inhibitor activity. Every ligand also has viral entry inhibitor activity, except for Hesperidin, whose probability of being active and inactive is unknown.

### 3.2. Molecular Docking Results

The docking scores and binding free energy are shown in Table 3 in descending order of the docking score performance (order binding free energies are shown in parenthesis). A lower docking score generated indicates a better binding mode of a protein-ligand complex. The docking score results show that Myricetin, Hesperidin, and Fisetin are the top three ligands as they have the lowest docking score, even lower than the lead compound Quercetin, while Galangin, Hesperetin, and Naringenin follow Quercetin.

The binding free energy generated by the MM/GBSA method is the free energy produced by the protein-ligand reaction to make bonds. This energy score is composed of various energies such as Coulomb energy, covalent binding energy, Van der Waals energy, lipophilic energy, generalized Born electrostatic solvation energy, hydrogen bonding energy, and *π*-*π* packing energy.

Like docking score, lower binding free energy indicates a better, more stable, and more favourable bond of complex protein-ligand. The result of binding free energy shows that Hesperidin has the lowest binding free energy, followed by Naringenin and Hesperetin.

Although Hesperidin shows strong performance in both docking score and MM/GBSA (Fisetin also in some degree shows strong performance in both docking score and MM/GBSA), Myricetin, Hesperetin, and Naringenin only show strong performance in either docking score or MM/GBSA but not both. Myricetin shows strong performance on docking score (#1) but meager on MM/GBSA (#6); on the other hand, Hesperetin and Naringenin show strong performance on MM/GBSA (#3 and #2, respectively) but meager on docking score (#6 and #7, respectively).

The difference between docking score and MM-GBSA values exists, and it is not supposed to be compared. Ignjatović et al. [24] found that there are almost no correlation between those two scoring functions, and both can be used independently as scoring function to predict binding affinity and ligands performance.

**Figure 3:**
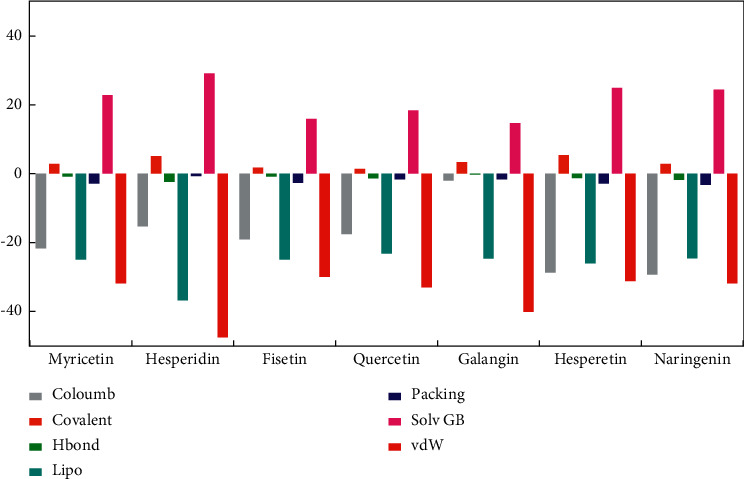
Contribution of different types of interactions in binding free energy of all protein-ligand complexes.

**Figure 4:**
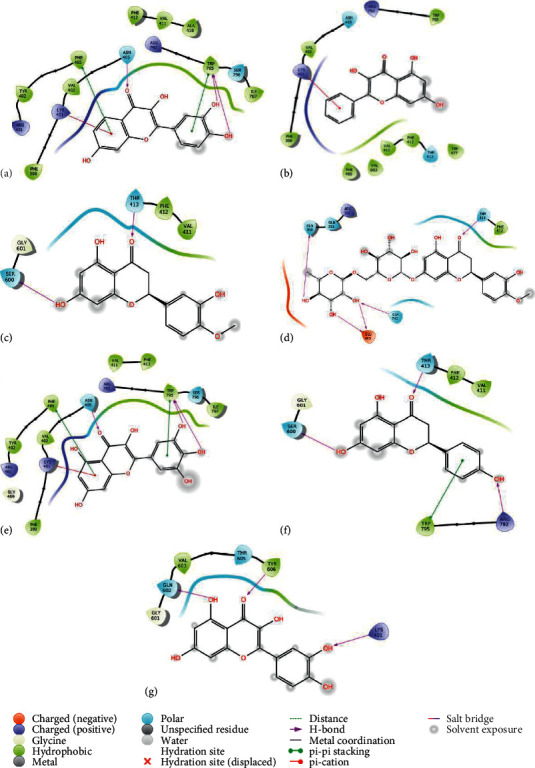
Ligand-protein 2D interaction diagram of molecular docking. (a) Fisetin, (b) Galangin, (c) Hesperetin, (d) Hesperidin, (e) Myricetin, (f) Naringenin, and (g) Quercetin.

The contribution of each type of bond for each protein-ligand complex's binding free energy is shown in Figure 3. Van der Waals energy is the main contributor for each protein-ligand complex, followed by Coulomb and lipophilic energy. Visualization of complex protein-ligand interaction was generated and shown as ligand interaction diagram with 3.00 Å of cut-off setting. The results show common interactions among all protein-ligand complexes such as hydrogen bonds, *π*-cation bond, and *π*-*π* stack. The ligand interaction diagram of all protein-ligand complexes is shown in Figure 4.

Fisetin (Figure 4(a)) forms a *π*-cation bond and two *π*-*π* stack interactions with amino acids Lys401, Phe485, and Trp795, respectively, and three hydrogen bonds: one with Asn405 (acceptor) and two with Trp795 (donors). There are parts of Fisetin that are exposed by solvent: all parts of the carbon ring, which has two hydroxyls, and a small part of the carbon ring, which has one hydroxyl. These interactions are supposed to give a good value of docking score, which Fisetin has.

Galangin (Figure 4(b)) only has one *π*-cation interaction to lys401 residue. Almost half of the Galangin is exposed to solvent, thus making other parts of Galangin closer to the active site and having hydrophobic interaction. The interaction is mainly due to this hydrophobic interaction. Therefore, it is expected that Galangin does not give strong interaction with the active site of protein RdRp.

Hesperetin (Figure 4(c)) forms two hydrogen bonds with Thr413 and Ser600. All the ends of carbon rings are exposed by solvent, thus forcing stringer interaction in the unexposed area of Hesperetin, which binds with Thr413. Polar and hydrophobic interactions will likely be dominant. As Galangin, Hesperetin does not give a strong performance on docking score.

On the other hand, Hesperidin (Figure 4(d)) forms five hydrogen bonds with residues of Gln350, Thr413, Glu463 (two bonds), and Gln742. Unlike Fisetin and Galangin, interactions established by Hesperidin are not driven by solvent exposure but primarily by charged abundant hydroxyl groups. Polar and hydrophobic interactions will not be as dominant as Fisetin and Galangin. This is supported by strong performance on both docking score and MM/GBSA.

Myricetin (Figure 4(e)) forms a *π*-cation interaction to Lys401, two *π*-*π* stack interactions to Phe485 and Trp795, and three hydrogen bonds to asn405 (acceptor) and two simultaneously to Trp795 (donor). If we look closely, Myricetin interactions to protein active site resemble Fisetin interactions protein. The similar molecular structure of Fisetin and Myricetin gives a similar interaction configuration, albeit it is stronger for Myricetin on docking score while Fisetin is stronger on MM/GBSA score.

Naringenin (Figure 4(f)) forms three hydrogen bonds to Thr413, Ser600 and Arg792, and *π*-*π* stack interaction to Trp795. Naringenin interactions with protein active site resemble to Hesperetin one. This is due to the similar molecular structure of Naringenin and Hesperetin, which does not give a a strong performance on docking scores. However, both give strong performance on MM/GBSA. The reason is that on MM/GBSA, various interactions were counted, including the incorporation of explicit terms for hydrophobic and solvation components, thus yielding higher scores for both Hesperetin and Naringenin in which hydrophobic interaction is likely dominant.

The last one is Quercetin, our control ligand (Figure 4(g)). It forms three hydrogen bonds to Gln602, Tyr606, and Lys401. Those three residues are binding sites according to both site mapping and database (Table 1). These interactions are likely driven by solvent exposure, which are not quite strong. As supported by docking score and MM/GBSA, the performance of Quercetin is not quite good.

### 3.3. ADMET Prediction Results

Table 4 shows a summarized prediction of toxicology and pharmacological properties of all ligands. It can be seen that all ligands are noncarcinogenic and nonmutagenic, predicted by Benigni/Bossa rules. All ligands also pass the AMES test indicating nontoxic properties. Fisetin, Galangin, Hesperetin, Naringenin, and Quercetin did not violate Lipinski's rule of five, indicating positive druglikeness of those compounds. Myricetin has six hydrogen bond donors, thus violating one Lipinski's rule, i.e., less than five hydrogen bonds, but still has positive druglikeness. Meanwhile, Hesperidin violates four Lipinski's rules, whose molecular mass exceeds 500 dalton, hydrogen bond donors and acceptors exceed the limit of 5 and 10, respectively, and molar refractivity is more than 130. Thus, Lipinski's rule of five eliminates the druglikeness of Hesperidin. However, we still further observed molecular dynamics of Hesperidin despite not obeying the Lipinski's rule of five, since those parameters are not a criterion for anchoring, instead, it is a filter for choosing molecule for druglikeness. The study of binding interaction by molecular dynamic and molecular docking does not require fulfilling Lipinski's rule of five [25].

### 3.4. Molecular Dynamics Result

The stability of docked complex and the binding pose obtained in docking studies are widely used to be verified by molecular dynamics simulation studies [20]. The molecular dynamic simulation yields Root Mean Square Deviation (RMSD), Root Mean Square Fluctuation (RMSF), and protein-ligand contact.

### 3.5. Root Mean Square Deviation (RMSD)

**Figure 5:**
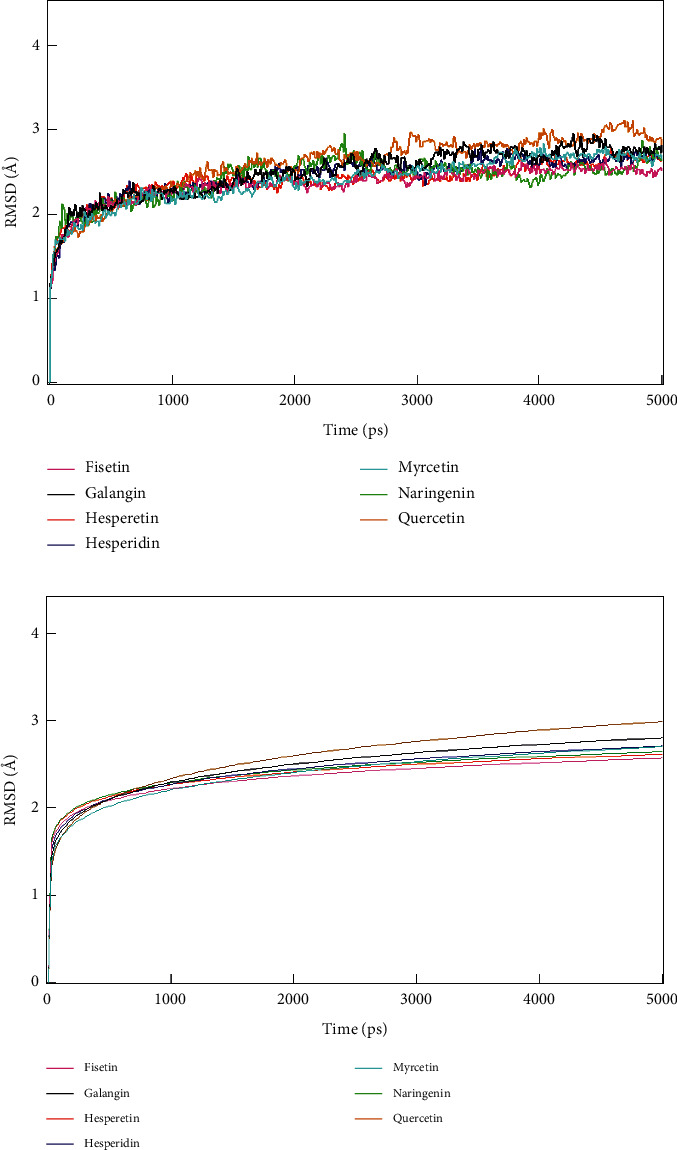
Root Mean Square Deviation (RMSD) of protein sidechain in molecular dynamics simulation. (a) True value of RMSD for a simulation time of 5000 ps. (b) Graph of the curve-fitting result of RMSD as power equation for a simulation time of 5000 ps.

RMSD of protein sidechain for each of each protein-ligand complex is shown in Figure 5(a). The stability of docking is observed by measuring the fluctuation of the RMSD value of each trajectory record every 9.6 ps (0.096 ns). RMSD value obtained from the molecular dynamics simulation process is determined based on the average displacement of a selected atom within a specific time frame. The cut-off interaction parameter was set to 10 Å for the ligand with the surrounding residues.

RMSD shows whether the system has reached stability by observing the fluctuation until the value is not significant enough after reaching a specific period. If the RMSD is still fluctuating significantly until a specific time, then the system is not stable. Fluctuations below 2.5 Å are acceptable for ligand-protein stable interaction. In contrast, fluctuations >3 Å indicate that the protein underwent a significant conformational change during the simulation, or it can be said that the protein is unstable [26].

The RMSD value within 0.01 ns has a sharp increase up to 2.2 Å due to ligand's effort to conform to the target. RMSD of all complexes reaches stability but at a different time. We further analyzed the RMSD data to validate the stability by curve-fitting analysis (Figure 5(b)). RMSD data was fit as the following power equation:

(2) $$\begin{array}{c} {\text{RMSD}}_{x}=A_{x}t^{n}, \end{array}$$

where *A*_*x*_ is constant, *t* is time (ps), and *n* is power coefficient. The higher *n* indicates a higher increase of RMSD value. The value of power coefficient *n* of RMSD for each protein-ligand complex is shown in Table 5. This result shows that Quercetin, the lead compound, has the highest power coefficient than the other ligands. It can be concluded that the other ligands yield a more stable interaction to RdRp.

### 3.6. Protein-Ligand Contact

**Figure 6:**
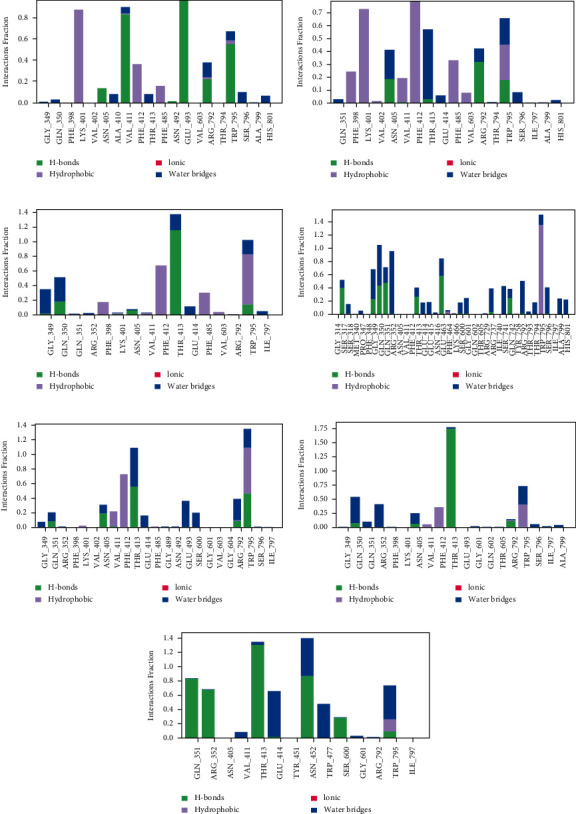
Protein-Ligand Contact of molecular dynamic simulation for 5000 ps. (a) Fisetin, (b) Galangin, (c) Hesperetin, (d) Hesperidin, (e) Myricetin, (f) Naringenin, and (g) Quercetin.

To validate the docking scores and MM-GBSA values, the dynamic analysis is conducted to observe the protein-ligand interaction for 5 ns to examine interaction consistency and location. The following analyses may quite diverge from interaction analyses given by molecular docking. Protein-ligand contact during the simulation was recorded and processed as a simulation interaction diagram and timeline representation. These results inform the type, fraction, which residue is involved, and when an interaction occurs in simulation time. In the simulation interaction diagram, the residue involved to interact with ligands is listed at the horizontal axis, and the type of interaction is informed by the colour indicator. The protein-ligand contact diagram of all ligand-protein complexes is shown in Figure 6.

Fisetin (Figure 6(a)) formed consistent interactions during the simulation period on the residues of Lys401, Val411, Glu493, and Trp795. The first former is given hydrophobic interaction and the three latter are given hydrogen bond interactions. This picture is quite different from the molecular docking result. However, it can be understood that hydrophobic interaction formed with Lys401 might be due to solvent exposure on Fisetin end part with the two hydroxyls carbon ring.

The protein-ligand interactions in Lys401 form hydrophobic bonds, while Val411 and Glu493 are due to hydrogen bonds and water bridges. However, from these consistent residues, some residues help stabilize the complex, namely, Phe412, Arg792, and Trp795, where hydrogen bonds and water bridges occur on the Arg792 and Trp795 residues, while in Phe412, the formation of hydrophobic interactions occurs. These residues are the binding site except for Glu493 that will not affect the inhibition process.

Galangin (Figure 6(b)) formed consistent interactions during the simulation period on the residues of Lys401, Phe412, Thr413, and Trp795. These residues ensure their essential role in making the complex more stable. Protein-ligand interactions that occur in Galangin are generally dominated by hydrophobic and water bridges. Some of the residues that also help the inhibition process are Asn405, Phe485, and Arg792. We found that the residues all bind at the binding site.

The complex of Hesperetin shows 16 residues contact (Figure 6(c)). Thr413 is residue with the highest fraction contact of 1,4, generated by hydrogen bonds and water bridges. A water bridge is essentially the same as a hydrogen bond; the water bridge is a hydrogen bond mediated by a water molecule, and the hydrogen bond geometry is slightly relaxed from the standard H-bond definition. Other residues that generate an appreciable fraction of contact are Gly349, Gln350, Phe412, Phe485, and Trp795. By discussing the binding site (Table 1), these residues are the binding site of RdRp.

The complex of Hesperidin shows significantly more contacts than the other, indicates good agreement with the molecular docking result. There is a total of 38 residues contact (Figure 6(d)). Trp795 is residue with the highest fraction contact of 1,5, generated by hydrophobic contact and water bridge. Hydrophobic contact falls into three subtypes: *π*-cation, *π*-*π*, and other nonspecific interactions. Other residues that generate an appreciable fraction of contact are Ser317, Gly349, Gln350, Gln351, Arg352, Thr413, Glu463, Arg737, Ser741, Gln742, Arg792, and Ser796. By discussing the binding site (Table 1), these residues are binding sites of RdRp, except Ser317 and Ser741.

Myricetin (Figure 6(e)) formed consistent interactions over the simulation period at the residues of Thr413 and Trp795. These residues ensure their essential role in inhibiting the RdRp enzyme. Myricetin has a much higher interaction fraction than Galangin or Fisetin. Protein-ligand interaction residues help slow the inhibition process but are not as good as the essential residues of Asn405, Val411, Phe412, Glu493, Ser600, and Arg792. Besides, Glu493 residue is a binding site residue, so the interaction between Myricetin and Glu493 residue is less affected in the inhibition process.

The complex of Naringenin shows 19 residues contact (Figure 6(f)). Thr413 is residue with the highest fraction contact of 1.75 generated by hydrogen bonds and water bridges. Other residues that generate an appreciable fraction of contact are Gln350, Gln351, Arg352, Asn405, Val411, Phe412, Arg792, Trp795, Ser796, and Ala799. By discussing the binding site (Table 1), these residues are the binding site of RdRp. The interaction fraction of the Naringenin complex is poorly distributed. One residue is dominant, while the others are minimal.

The complex of Quercetin shows 14 residues contact (Figure 6(g)). Asn452 is residue with the highest fraction contact of 1.4, generated by hydrogen bonds and water bridges. Other residues that cause an appreciable fraction of contact are Gln351, Arg352, Thr413, Glu414, Trp477, Ser600, and Trp795. By discussing the binding site (Table 1), these residues are the binding site of RdRp. In contrast to the complex of Naringenin, Quercetin shows a fair distribution of fraction interaction.

### 3.7. RMSF Protein-Ligand Contact

**Figure 7:**
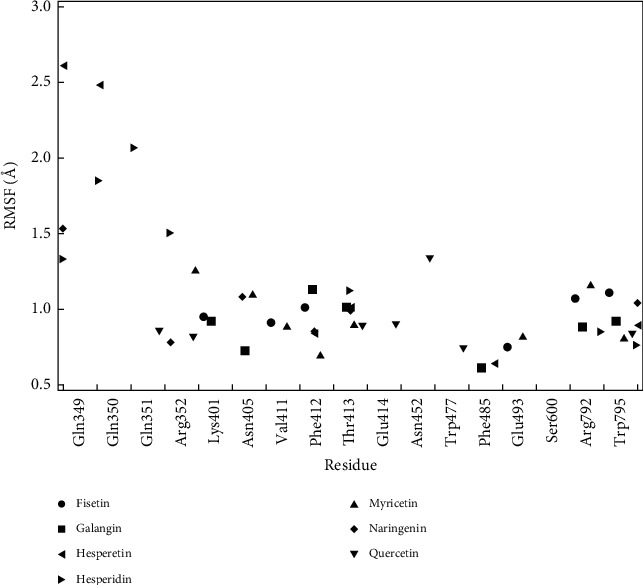
RMSF of protein-ligand contact.

In validating protein-ligand contact stability, we observe the Root Mean Square Fluctuation (RMSF) of residues where interaction between ligands and target protein occurred (Figure 7). RMSF is a local fluctuation of each residue in the protein, and by this data, we can observe whether an individual residue of protein target was stable during the simulation. The residue chosen is those with significant fraction interaction of protein-ligand contact. The threshold of 2,5 Å is set to mark the relatively high value of RMSF.

Overall, RMSF of protein-ligand contact shows a low value of RMSF, which indicates stable contact at the residue. Only two residues of the Hesperetin-protein complex show a high value of RMSF, namely, Gln349 and Gln350. Even though those two residues are not the highest interaction fraction of complex Hesperetin, this shows that the complex is not so stable as the other ligands.

## 4. Conclusions

In this study, the interaction models of DENV-3-RdRp with Fisetin, Galangin, Hesperetin, Hesperidin, Myricetin, and Naringenin were observed by molecular docking and molecular dynamic simulation. The results are compared with those Quercetin as the well-known ligand inhibitor of DENV RdRp. QSAR analysis indicates a positive result of the antiviral activity for all ligands. Docking score and MM/GBSA gave quite different results where Myricetin, Hesperidin, and Fisetin show strong performance in docking score; on the other hand, Hesperidin, Hesperetin, and Naringenin show strong performance in MM/GBSA. Only Hesperidin shows strong performance in both scorings. Our molecular dynamics study through RMSD showed that even though Quercetin does not give good scoring values in both docking score and MM/GBSA, it has robust stable interaction to RdRp. The strong performance of Hesperidin was also validated by protein-ligand contact fraction in 5 ns. RMSF results showed that only Hesperetin does not perform well compared to other investigated ligands. Overall, we observed that Hesperidin shows good potential as a DENV-3-RdRp inhibitor well beyond Quercetin, despite not obeying Lipinski's rule of five. Further in vitro study should be conducted to validate this found.

## Figures and Tables

**Table 1:** Binding site residue of DENV-3 Rdrp.

Binding site residue obtained from sitemap								
302	318	339	340	341	343	345	346	347
348	349	350	351	352	355	356	357	358
398	401	402	405	408	411	412	413	414
451	452	453	457	458	460	463	477	481
485	489	492	511	531	532	533	534	535
536	537	538	539	575	597	598	599	600
601	603	605	606	609	661	662	663	664
665	688	689	691	695	696	697	698	700
707	708	709	710	711	729	733	734	737
738	740	742	758	761	762	765	766	792
793	794	795	796	797	798	799	800	801
802	803							
*Native ligand*	Residue binding site obtained from database.							
PEG	822	823						
VWS	401	405	411	412	413	481	485	492
	600	601	602	603	604	605	606	795
	797							
ZN	437	441	446	449	712	714	728	847

**Table 2:** PASS prediction of ligands activity.

Activity		Antiviral	Viral entry inhibitor	RNA synthesis inhibitor
Fisetin	Pa	0.251	0.275	0.32
	Pi	0.059	0.014	0.043

Galangin	Pa	0.266	0.256	0.344
	Pi	0.051	0.028	0.033

Hesperetin	Pa	0.164	0.328	0.397
	Pi	0.143	0.004	0.02

Hesperidin	Pa	0.193	—	0.587
	Pi	0.102	—	0.003

Myricetin	Pa	0.334	0.272	0.322
	Pi	0.026	0.016	0.042

Naringenin	Pa	0.197	0.309	0.393
	Pi	0.098	0.005	0.021

Quercetin	Pa	0.262	0.257	0.345
	Pi	0.053	0.027	0.033

**Table 3:** Docking scores and MM-GBSA binding free energies.

No	Ligand	*Docking score* (Kcal/mol)	Binding energy (Kcal/mol)
1 (6)	Myricetin	−10.145	−57.19
2 (1)	Hesperidin	−9.842	−69.31
3 (4)	Fisetin	−9.796	−60.65
4 (5)	Quercetin	−8.513	−57.83
5 (7)	Galangin	−8.036	−51.36
6 (3)	Hesperetin	−7.761	−60.93
7 (2)	Naringenin	−5.634	−64.90

Note. Number reflects order for docking score (number in parentheses reflects for MM/GBSA).

**Table 4:** Predicted toxicology and pharmacological properties by ADMET analysis.

Ligand	Benigni/Bossa rules		AMES test	Lipinski's rule of five		Skin corrosive
	Carcinogenicity	Mutagenicity		Violation	Druglikeness	
Fisetin	Noncarcinogenic	Nonmutagenic	Pass	0	Yes	No
Galangin	Noncarcinogenic	Nonmutagenic	Pass	0	Yes	No
Hesperetin	Noncarcinogenic	Nonmutagenic	Pass	0	Yes	No
Hesperidin	Noncarcinogenic	Nonmutagenic	Pass	4	No	Yes
Myricetin	Noncarcinogenic	Nonmutagenic	Pass	1	Yes	No
Naringenin	Noncarcinogenic	Nonmutagenic	Pass	0	Yes	No
Quercetin	Noncarcinogenic	Nonmutagenic	Pass	0	Yes	No

**Table 5:** Power coefficient of RMSD.

Ligand	Power coefficient (*n*)
Fisetin	0.09
Galangin	0.13
Hesperetin	0.08
Hesperidin	0.10
Myricetin	0.13
Naringenin	0.09
Quercetin	0.15

## Data Availability

The data that support the findings of this study are available from the corresponding author upon reasonable request.
